# Supplementary material for: Disarming emotional memories using targeted memory reactivation during rapid eye movement sleep
Source: Imaging Neurosci (Camb). 2025 Oct 10;3:IMAG.a.924. doi: 10.1162/IMAG.a.924 (PMC12516159; doi:10.1162/IMAG.a.924)
Supplement: Supplementary Material [file IMAG.a.924_supp.pdf]

Supplemental Information

Disarming emotional memories using Targeted Memory reactivation during Rapid-Eye movement sleep

Viviana Greco[1], Tamas A. Foldes[1], Neil A. Harrison[1], Kevin Murphy[2], Marta Wawrzuta[1], Mahmoud E. A. Abdellahi[1], Penelope A. Lewis[1*]

[1]Psychology Department, Cardiff University Brain Research Imaging Centre, (CUBRIC), Cardiff University, Cardiff, UK

[2]School of Physics, Cardiff University Brain Research Imaging Centre (CUBRIC), Cardiff University, Cardiff, UK

*Lewis A. Penelope

lewisp8@cardiff.ac.uk

Supplemental figures

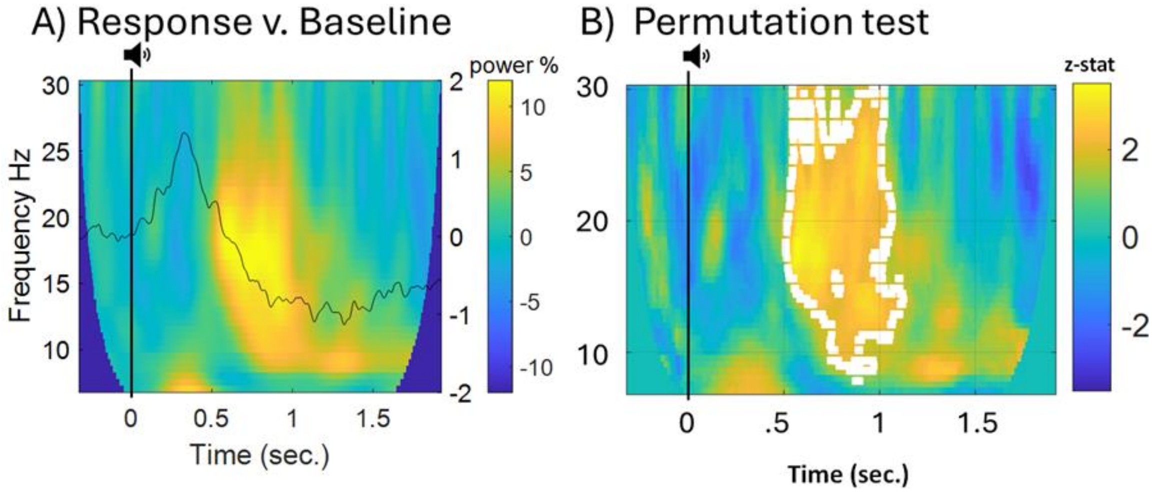

Figure S1. ERP and time-frequency analyses, A) ERP analysis using baseline duration of 400 ms pre-stimulus and time-frequency analysis measured as percentage change from baseline (400 ms pre-stimulus). B) Correction of multiple comparisons using cluster-based permutation for the time-frequency analysis showed a significant effect explained by a cluster that spans frequencies from around 10Hz to 30Hz in the duration from 0.5sec. to 1 sec. ($n = 16$, $p = 0.0079$).

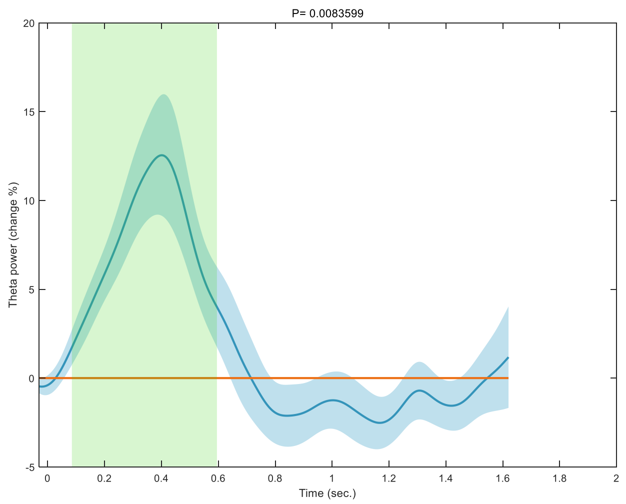

Figure S2. Theta specific power analysis. This shows that Theta power was significantly higher than baseline duration [-400ms 0] during ~0.1 - 0.6 seconds post cue.

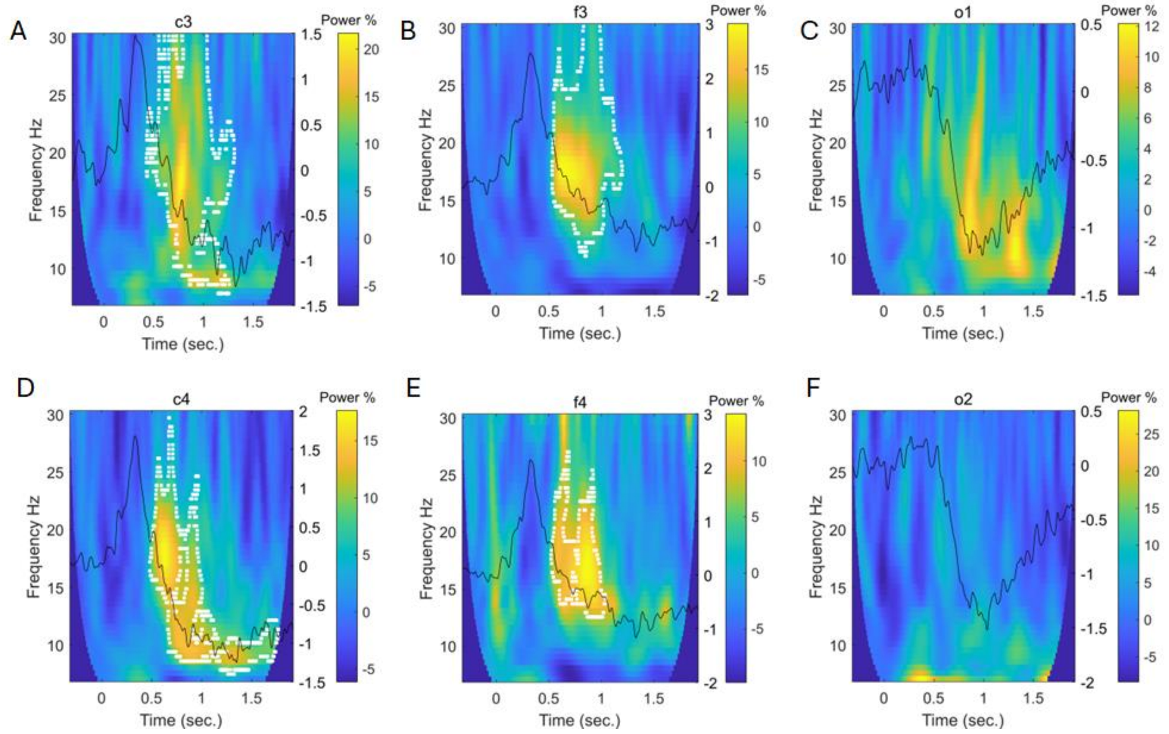

Figure S3. ERP and Time frequency decomposition on every EEG channel. This shows a consistent power increase among channels compared to baseline duration [-400ms 0] except for the two occipital channels (O1 and O2). Significant responses are outlined in white.

Supplementary Tables

Sleep data	Mean ± SD
TST (min)	528.91 ± 37.38
W (min)	43.47 ± 39.05
N1 (min)	26.31 ± 14.20
N2 (min)	222.84 ± 39.85
SWS (min)	128.34 ± 44.23
REM (min)	95.09 ± 27.42

Table S1. Sleep parameters. Time spent in each sleep stages is reported in minutes and as percentage of total sleep time (mean ± SD). TST = total sleep time; W = wake; N1 = NREM Stage 1; N2 = NREM Stage 2; N3 = NREM Stage 3; REM = Rapid Eye Movement Sleep. N = 16.

**A) LME model results**					
Predictors	*Model Estimates*	*CI*	*p*		
Intercept	3.03	2.68 – 3.38	**<0.001**		
Cueing (Cued)	-0.04	-0.15 – 0.07	0.457		
Baseline	0.43	0.37 – 0.49	**<0.001**		
Session (2-Wk)	-0.29	-0.41 – -0.17	**<0.001**		
Cueing (Cued) x Baseline	-0.10	-0.17 – -0.02	**0.010**		
Session (2-Wk)	0.05	-0.12 – 0.21	0.564		
**B) Post-hoc analysis**					
	Model Estimate	*SE*	*df*	*t.stat*	*p*
Baseline = -2					
Uncued > Cued	-0.18	0.09	1518	2.02	**0.04**
Baseline = -1					
Uncued > Cued	-0.08	0.06	1519	1.38	**0.17**
Baseline = 0					
Uncued > Cued	0.02	0.04	1515	-0.42	0.67
Baseline = 1					
Uncued > Cued	0.11	0.06	1511	-2.04	**0.04**
Baseline = 2					
Uncued > Cued	0.21	0.09	1511	-2.46	**0.01**

Table S2. A) Results of the mixed effect model examining the effect of Cueing (Cued/Uncued), Sessions (Session 48-H /Session 2-Wk) and pre-sleep Baseline on subjective arousal ratings. Model Estimates column lists regression coefficients. For each line, the reference value is

indicated in brackets (e.g. Cueing (cued)). Notably the significant intercept indicates the predicted mean value of the model when all parameters and conditions are at reference level. B) post-hoc analysis of items with each mean baseline rating (-2 to 2), showing the basis of the interaction from (A).

	**Mean**		**Standard Error (SE)**	
	**Cued**	**Uncued**	**Cued**	**Uncued**
**Session 1 (S1)**	3.04	3.08	0.05	0.05
**Session 2 (S2) – 48h after S1**	2.97	3.04	0.06	0.05
**Session 3 (S3) – 2 weeks after S1**	2.83	2.85	0.06	0.05

Table S3. Descriptive statistic of the Arousal Rating Task.

		**Cued**			**Uncued**		
		Pearson's correlation	p-value	p-value (FDR corr)	Pearson's correlation	p-value	p-value (FDR corr)
Ratings S2	Insula	0.23	0.36	0.54	0.06	0.80	0.80
	OFC	0.18	0.49	0.56	0.15	0.56	0.56
	sgACC	-0.01	0.97	0.97	-0.20	0.44	0.66
	Amygdala	0.10	0.70	0.70	0.38	0.12	0.36
HRD	Insula	-0.09	0.75	0.75	0.33	0.25	0.33
	OFC	-0.25	0.38	0.38	0.36	0.20	0.27
	sgACC	-0.63	0.02	0.08	0.15	0.62	0.62
	Amygdala	0.14	0.63	0.63	0.39	0.17	0.23

Table S4. Correlations between mean parameter estimates and behavioural results or

HRD. Results of Pearson's correlations between the mean parameter estimates for each subject in our ROIs, behavioral results in S2, and heart rate deceleration (HRD). Both the uncorrected and FDR-corrected p-values are reported.

		Cued			Uncued		
		Pearson's correlation	p-value	p-value (FDR corr)	Pearson's correlation	p-value	p-value (FDR corr)
HRD	TST (min)	0.01	0.95	0.97	-0.09	0.76	0.94
	N2 (%)	0.21	0.44	0.97	0.06	0.85	0.94
	N3 (%)	-0.09	0.75	0.97	-0.06	0.84	0.94
	REM (%)	0.24	0.37	0.97	0.19	0.52	0.94
HRD	Ratings S2	0.07	0.81	0.82	0.13	0.65	0.73

Table S5. Correlations between HRD, sleep, and behavioural measurements. Results of Pearson's correlations between HRD for cued and uncued image-sound pairs and the percentage of time spent in each sleep stage, total sleep time, and behavioural results in S2. Both the uncorrected and FDR-corrected p=values are reported.

	AIC	BIC	Chisq	df	Pr(>Chisq)
Model 1	4260.83	4298.32			
Model 2	4001.07	4043.92	261.75	1	0.00
Model 3	3996.48	4044.68	6.60	1	0.01

Table S6. Model comparison. AIC = Akaike Information Criterion; BIC = Bayesian Information Criterion are both measures of the relative quality of statistical models. Chisq = Chi-squared, test statistic for the LRT, showing how each model compares to the one directly before (Model 2 vs Model 1; Model 3 vs. Model 2).

Slide No.	Description	Valence (M±SD)	Arousal (M±SD)
1112	Snake	4.71(1.70)	4.60(2.44)
1300	PitBull	3.55(1.78)	6.79(1.84)
1930	Shark	3.79(1.92)	6.42(2.07)
2095	Toddler	1.79(1.18)	5.25(2.34)
2717	DrugAddict	2.58(1.32)	5.70(2.16)
2799	Funeral	2.42(1.41)	5.02(1.99)
2800	CryingBoy	2.45(1.42)	5.09(2.15)
3016	Mutilation	1.90(1.31)	5.82(2.44)
3051	Mutilation	2.30(1.86)	5.62(2.45)
3068	Mutilation	1.80(1.56)	6.77(2.49)
3170	BabyTumor	1.46(1.01)	7.21(1.99)
3210	Surgery	4.49(1.91)	5.39(1.91)
3215	BurnVictim	2.51(1.32)	5.44(2.16)
3225	Mutilation	1.82(1.22)	5.95(2.46)
3230	Dyingman	2.02(1.30)	5.41(2.21)
3261	Tumor	1.82(1.34)	5.75(2.64)
3301	InjuredChild	1.80(1.28)	5.21(2.26)
6000	Prison	4.04(1.74)	4.91(2.17)
6021	Assault	2.21(1.51)	6.06(2.38)
6242	Gang	2.69(1.59)	5.43(2.36)
6315	BeatenFem	2.31(1.69)	6.38(2.39)
6550	Attack	2.73(2.38)	7.09(1.98)
6560	Attack	2.16(1.41)	6.53(2.42)
6834	Police	2.91(1.73)	6.28(1.90)
8485	Fire	2.73(1.62)	6.46(2.10)
9000	Cemetery	2.55(1.55)	4.06(2.2.5)

9041	ScaredChild	2.98(1.58)	4.64(2.26)
9050	PlaneCrash	2.42(1.61)	6.36(1.97)
9230	OilFire	3.89(1.58)	5.77(2.36)
9252	DeadBody	1.98(1.59)	6.64(2.33)
9253	Mutilation	2.00(1.19)	5.53(2.40)
9254	Assault	2.03(1.35)	6.04(2.35)
9301	Toilet	2.26(1.56)	5.28(2.46)
9320	Vomit	2.65(1.92)	4.93(2.70)
9405	SlicedHead	1.83(1.17)	6.08(2.40)
9410	Soldier	1.51(1.15)	7.07(2.06)
9420	Soldier	2.32(1.59)	5.69(2.28)
9423	Assault	2.61(1.51)	5.66(2.15)
9424	Bomb	2.87(1.62)	5.78(2.12)
9425	Assault	2.67(1.44)	5.92(2.13)
9429	Assault	2.68(1.26)	5.63(2.04)
9571	Cat	2.46(1.61)	5.41(2.27)
9582	DentalExam	4.18(2.28)	5.29(2.21)
9622	Jet	3.10(1.90)	6.26(1.98)
9630	Bomb	2.96(1.72)	6.06(2.22)
9800	Skinhead	2.04(1.57)	6.05(2.71)
9900	CarAccident	2.46(1.39)	5.58(2.13)
9921	Fire	2.04(1.47)	6.52(1.94)

Table S7. 48 standardized images taken from the IAPS database. Overall valence = Mean ± SD, 2.57 ± 0.75; overall arousal = Mean ± SD, 5.81 ± 0.68.
